# Long‐Term Mortality in Diabetic Foot Disease is Higher Than That of Overall Cancer: Results From Three International Cohorts

**DOI:** 10.1002/dmrr.70221

**Published:** 2026-09-08

**Authors:** Qinglian Zeng, Ziwei Tang, Xiangjun Chen, Linlin Zhan, Victoria Balasubramanian, Zhihao Chen, Manman Du, Qi Zhang, Fei Lian, Xiaoru Zhang, Qing Liang, Wenrui Zhao, Xun Li, Mengyao Zhang, Qifu Li, Shumin Yang, Jinbo Hu, Qingfeng Cheng, Hang Shen

**Affiliations:** ^1^ Department of Endocrinology Sichuan‐Chongqing Joint Key Laboratory of Metabolic Vascular Diseases Chongqing Key Laboratory of Translational Medicine in Major Metabolic Diseases The First Affiliated Hospital of Chongqing Medical University Chongqing China; ^2^ Diabetes Research Centre University of Leicester Leicester General Hospital Leicester UK

**Keywords:** diabetic foot disease, mortality risk, overall cancer, site‐specific cancer

## Abstract

**Background:**

Guidelines suggest that the risk of mortality in diabetic foot disease (DFD) is comparable to that of cancer; however, high‐quality evidence is lacking. We aimed to compare the mortality of DFD with that of various cancers based on data from international cohorts.

**Methods:**

We defined DFD according to the IWGDF 2023 guidelines, and cancer was defined based on the ICD‐10 codes. We included 527, 1784, and 601 patients with DFD and 1219, 100,643, and 152 patients with cancer from the National Health and Nutrition Examination Survey (NHANES), UK Biobank (UKB), and Chongqing Diabetes Registration (CDR) cohorts, respectively. We compared the risk of mortality between DFD and overall or site‐specific cancers in the same cohort.

**Results:**

Patients with DFD showed 38%, 45%, and 499% higher risks of mortality than patients with overall cancer in the NHANES, UKB, and CDR. For site‐specific cancer, the risk of mortality was significantly higher in DFD than in skin cancer (hazard ratio 10.69, 95% CI: 1.37–2.07 in NHANSE; 3.8, 3.72–448 in UKB), breast cancer (1.35, 1.0–1.83 in NHANSE; 1.95, 1.67–227 in UKB), and prostate cancer (1.4, 1.8–179 in NHANSE; 2.61, 2.35–289 in UKB). The mortality rate of DFD is similar to that of most types of cancer, such as colon cancer, lymphoma, and kidney cancer, and is lower than that of a minority of cancers, including lung, pancreatic, and liver cancers.

**Conclusion:**

Patients with DFD have a higher risk of mortality than overall cancer, particularly skin, breast, and prostate cancers.

## 1 Introduction

Diabetic foot disease (DFD) is defined as the presence of foot‐specific complications in individuals with diabetes, including peripheral neuropathy, peripheral arterial disease, and foot ulceration, according to the 2023 International Working Group on the Diabetic Foot criteria [1]. DFD represents a particularly devastating and costly complication, often leading to prolonged hospitalisation, amputations, and associated with a markedly elevated mortality risk, with a 5‐year mortality rate of 30%–50% [2, 3, 4]. To contextualise the severity of this mortality risk and elevate DFD's recognition as a critical public health concern, its mortality burden is often benchmarked against other highly lethal conditions, notably various cancers. Several guidelines and scientific statements suggest that its mortality risk is comparable to or exceeds that of cancers such as breast or colon cancer [5, 6, 7]. However, high‐quality evidence for this claim is lacking.

There is a significant research gap in the direct comparison of mortality risk between DFD and cancer. The aforementioned guidelines and statements often cite evidence from commentaries. By comparing the reported mortality data for DFD and cancers, this commentary suggested that the mortality risk of DFD is comparable to the pooled 5‐year mortality rate for all reported cancers, exceeding that of breast cancer but remaining lower than that of lung cancer [8]. Crucially, since the mortality data for DFD and cancers were derived from distinct populations, this compromises the generalisability and reliability of the conclusion. Further studies that directly compare DFD mortality with overall and specific cancers within the same cohort are needed.

To overcome these critical limitations and generate the high‐quality evidence necessary for robust clinical guidelines and public health statements, our study was designed to directly compare the mortality risk between DFD and overall or site‐specific cancers within the same patient cohorts. Furthermore, by utilising three independent, multi‐ethnic populations, we aimed to validate these findings and ensure broader generalisability, thereby providing a more accurate quantification of DFD's disease burden and its implications for public health.

## 2 Methods

### 2.1 Study Population

This study included three international cohorts: the National Health and Nutrition Examination Survey (NHANES), the UK Biobank (UKB), and the Chongqing Diabetes Registry (CDR). The NHANES is a nationally representative, biennial survey designed to assess the health and nutritional status of the U.S. population. The survey protocols were approved by the Centres for Disease Control and Prevention National Centre for Health Statistics Ethics Review Board, and written informed consent was obtained from all the participants. As only de‐identified publicly available data were used in this analysis, additional institutional review board approval was not required for this study.

The UKB is a population‐based prospective cohort that recruited 502,241 participants aged 40–69 years between 2006 and 2010 from the United Kingdom. The present analysis was approved by the UKB (Application ID 103654) [9, 10]. The UK Biobank cohort received approval from the National Information Governance Board for Health and Social Care and the National Health Service North West Multicenter Research Ethics Committee (reference 13/NW/0382). All participants provided electronic informed consent for the study's procedures.

The CDR is a prospective cohort established in 2017 that enrolled 3956 patients with type 2 diabetes in Chongqing, China (ClinicalTrials.gov Identifier: NCT03692884) [11]. Diabetes was diagnosed according to the 1999 World Health Organization criteria. Participants were followed up regularly with detailed assessments of biochemical markers and documentation of renal and cardiovascular outcomes. The study was approved by the Ethics Committee of the First Affiliated Hospital of Chongqing Medical University, and written informed consent was obtained from all participants.

### 2.2 Assessment of DFD

The definition of DFD was based on the 2023 International Working Group on the DFD (IWGDF) criteria, incorporating peripheral artery disease, foot ulceration or neuropathy [1]. Lower extremity artery disease was defined as an ankle‐brachial index < 0.9 or > 1.4 in either limb, or identified by ICD‐10 codes R02 and I7021, OPCS‐4 procedure codes L591‐L599, L601‐L604, L631, and L635, self‐reported diagnoses (codes 1087, 1440‐1442, 1102, and 1108), or claudication based on the WHO questionnaire. Lower extremity ulceration was defined as a self‐reported open wound or gangrene on the foot or identified using ICD‐10 code L97, excluding venous ulcers (L83). Lower extremity neuropathy was defined as at least one insensate area on the plantar surface of the foot using the 10‐g monofilament test (three sites were tested). A total of 572, 1691, and 678 DFD cases were enrolled from the NHANES, UKB, and CDR, respectively.

### 2.3 Assessment of Cancer

In the UKB, cancer was defined according to ICD‐10 codes C00‐C97, representing malignant neoplasms and excluding secondary cancers (Supporting Information S1: Table S1). For harmonisation, ICD‐10 codes were subsequently mapped to the cancer categories used in the NHANES questionnaires. In the NHANES, cancer status was determined through self‐reported surveys along with the specific cancer type. In the CDR, cancer was defined based on physician‐diagnosed malignancy recorded during hospitalisation. A total of 1219, 100,736, and 152 participants with cancer were included in the NHANES, UKB, and CDR, respectively.

### 2.4 Statistical Analysis

Categorical variables were expressed as percentages, and continuous variables were presented as mean (standard deviation), with group comparisons conducted using the Student's *t*‐test or the Mann–Whitney *U* test. Participants were followed from the date of cohort entry until the occurrence of all‐cause mortality, the date of last contact, or the study‐specific cutoff date, whichever came first. The cutoff dates were December 2019 for the NHANES, October 2022 for the UKB, and March 2024 for the CDR. Individuals who did not experience the outcome of interest were censored at their last available follow‐up date. We applied Cox proportional hazard models to assess the mortality risk of patients with DFD versus those with any cancer or site‐specific cancers. Multivariable models were constructed to adjust for potential confounders, including age, sex, and body mass index (BMI). Hazard ratios (HRs) with 95% confidence intervals (CIs) were reported for the analyses. A heatmap was generated to visualise the differences in overall and site‐specific mortality rates between DFD and cancer in the NHANES and UK Biobank cohorts. The *p* < 0.05 was considered statistically significant. Data were analysed using R 4.0.3 (R Foundation for Statistical Computing, Vienna, Austria). We conducted all statistical analyses on a supercomputer platform (Inspur M5).

## 3 Results

### 3.1 Clinical Characteristics of Participants

The baseline characteristics of participants with cancer, diabetes without DFD, or DFD across the NHANES, UKB, and CDR cohorts are detailed in Table 1. Compared to participants with either cancer or diabetes without DFD, those with DFD exhibited a higher proportion of males, higher body mass index, glycated haemoglobin, low‐density lipoprotein cholesterol, and serum creatinine, a lower estimated glomerular filtration rate, and an increased proportion of individuals with heart failure.

**TABLE 1:** Characteristics of participants with cancer, diabetes alone, and diabetic foot disease across three cohorts.

	NHANES			UKB			CDR		
	Cancer	Diabetes	DFD	Cancer	Diabetes	DFD	Cancer	Diabetes	DFD
No.	1219	625	572	100736	36130	1691	152	3422	678
Age (years)	66.9 (15.5)	60.3 (10.9)	66.5 (11.4)	60.0 (7.0)	58.2 (7.6)	60.0 (7.0)	63.4 (8.4)	57.0 (12.2)	64.9 (11.9)
Male	577 (47.3)	329 (52.6)	337 (58.9)	49742 (49.4)	20701 (57.4)	1324 (74.2)	71 (46.7)	2032 (59.4)	452 (66.7)
BMI (kg/m^2^)	27.7 (5.8)	30.5 (5.4)	31.2 (6.7)	27.6 (4.8)	31.3 (5.8)	32.3 (6.3)	24.5 (3.4)	24.6 (3.6)	23.3 (3.4)
Plasma glucose (mmol/L)	5.8 (1.3)	8.9 (3.7)	9.7 (4.2)	5.2 (1.3)	6.6 (2.9)	8.3 (4.5)	8.6 (2.9)	9.0 (3.4)	7.4 (2.1)
HbAlc (%)	5.5 (0.6)	7.4 (1.8)	7.6 (1.9)	5.5 (0.6)	6.5 (1.2)	7.3 (1.7)	8.4 (2.1)	9.2 (2.5)	9.7 (2.7)
LDL‐C (mmol/L)	3.1 (0.9)	3.1 (0.9)	2.9 (0.9)	3.5 (0.9)	3.1 (0.9)	2.8 (0.9)	2.5 (1.0)	2.5 (1.0)	2.3 (1.0)
Serum creatinine (mmol/L)	79.6 (61.9, 97.2)	70.7 (61.9, 88.4)	79.6 (61.9, 106.1)	71.6 (62.0, 82.4)	71.9 (62.0, 83.0)	74.5 (63.2, 89.8)	64.0 (54.0, 82.8)	66.0 (55.0, 81.0)	78.0 (60.0, 112.0)
eGFR (mL/min/1.73 m^2^)	76.8 (63.6, 96.1)	86.3 (72.5, 107.2)	76.4 (55.8, 99.6)	89.6 (79.0, 101.1)	92.5 (80.1, 106.2)	90.7 (73.3, 107.9)	108.5 (81.4, 137.8)	110.8 (88.9, 135.9)	88.9 (57.7, 122.5)
Heart failure	92 (7.5)	29 (4.6)	77 (13.5)	748 (0.7)	1242 (3.4)	345 (20.4)	4 (2.6)	49 (1.4)	39 (5.7)

*Note:* Continuous variables were expressed as mean (standard deviation) or median (25th and 75th percentiles), and categorical variables were expressed as *n* (%).

Abbreviations: BMI, body mass index; CDR, Chongqing Diabetes Registry; DFD, diabetic foot disease; eGFR, estimated glomerular filtration rate; HbA1c, haemoglobin A1c; LDL‐C, low density lipoprotein cholesterol; NHANES, National Health and Nutrition Examination Survey; UKB, UK Biobank.

### 3.2 Mortality in Patients With DFD Versus Overall Cancers

**FIGURE 1:**
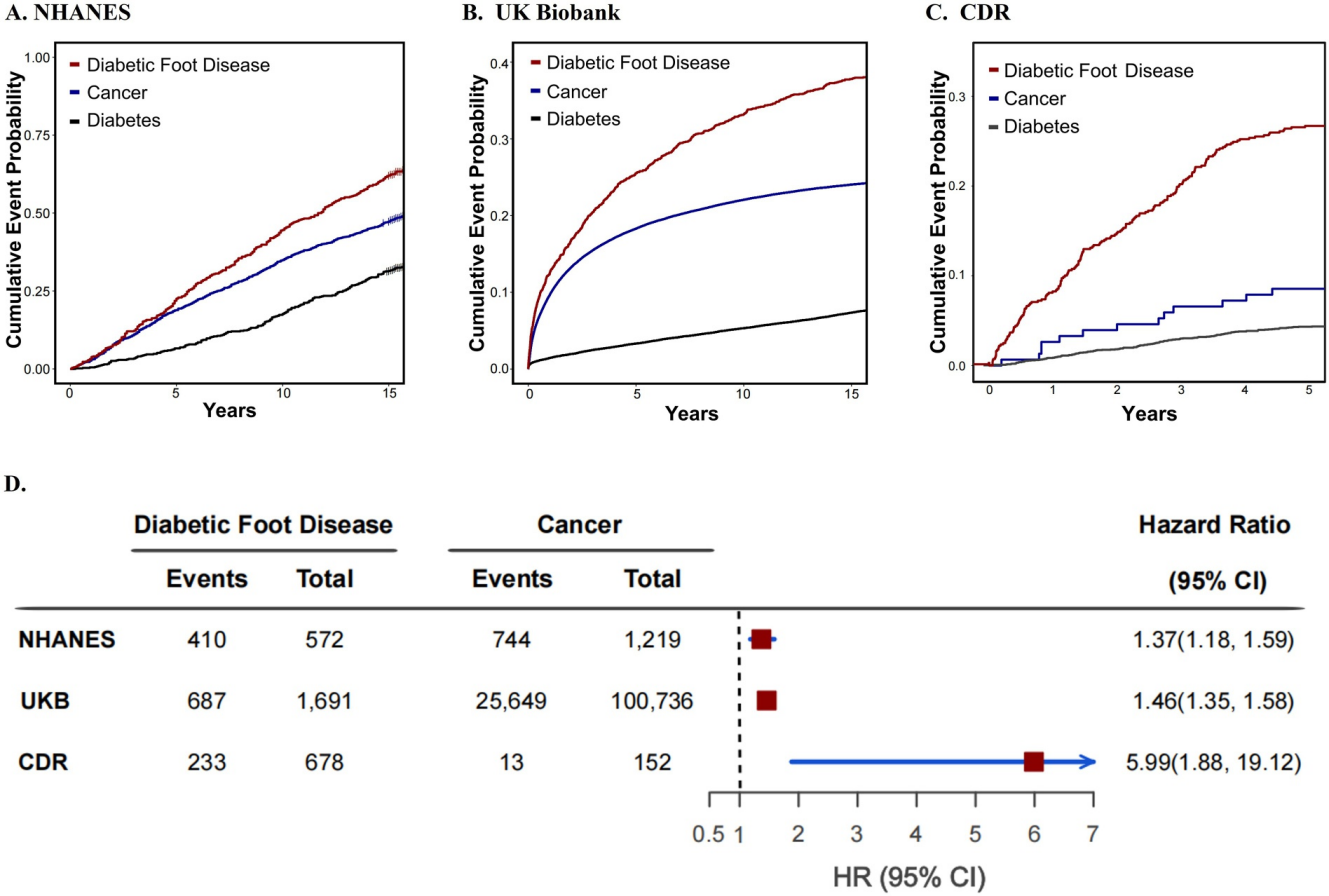
Mortality in patients with diabetic foot disease versus overall cancer. (A–C) Kaplan–Meier curves of mortality among patients within three cohorts. (D) Forest plot for mortality risk comparison between diabetic foot disease and overall cancer in three cohorts.

After a median follow‐up of 17.0, 13.3, and 4.0 years, the all‐cause mortality rates were 58.4%, 22.0%, and 9.4% in the NHANES, UKB, and CDR cohorts, respectively. Across all cohorts, overall mortality was higher in patients with DFD than in those with cancer and in diabetic patients without DFD (NHANES: 71.7% vs. 61.0% vs. 41.4%; UKB: 40.6% vs. 25.5% vs. 11.4%; CDR cohort: 34.4% vs. 8.6% vs. 4.4%) (Supporting Information S1: Table S2). When the 1‐, 5‐, and 10‐year mortality rates were analysed separately, DFD mortality was consistently higher than cancer mortality across the three cohorts (Supporting Information S1: Table S2). Compared with participants with cancer, those with DFD had a significantly increased risk of mortality (NHANES: HR 1.38, 95% CI: 1.19–1.60; UKB: HR 1.45, 95% CI: 1.34–1.56; CDR: HR 5.99, 95% CI: 1.88–19.12) (Figure 1A–D, Supporting Information S1: Table S3).

### 3.3 Mortality in Patients With DFD Versus Site‐Specific Cancers

**FIGURE 2:**
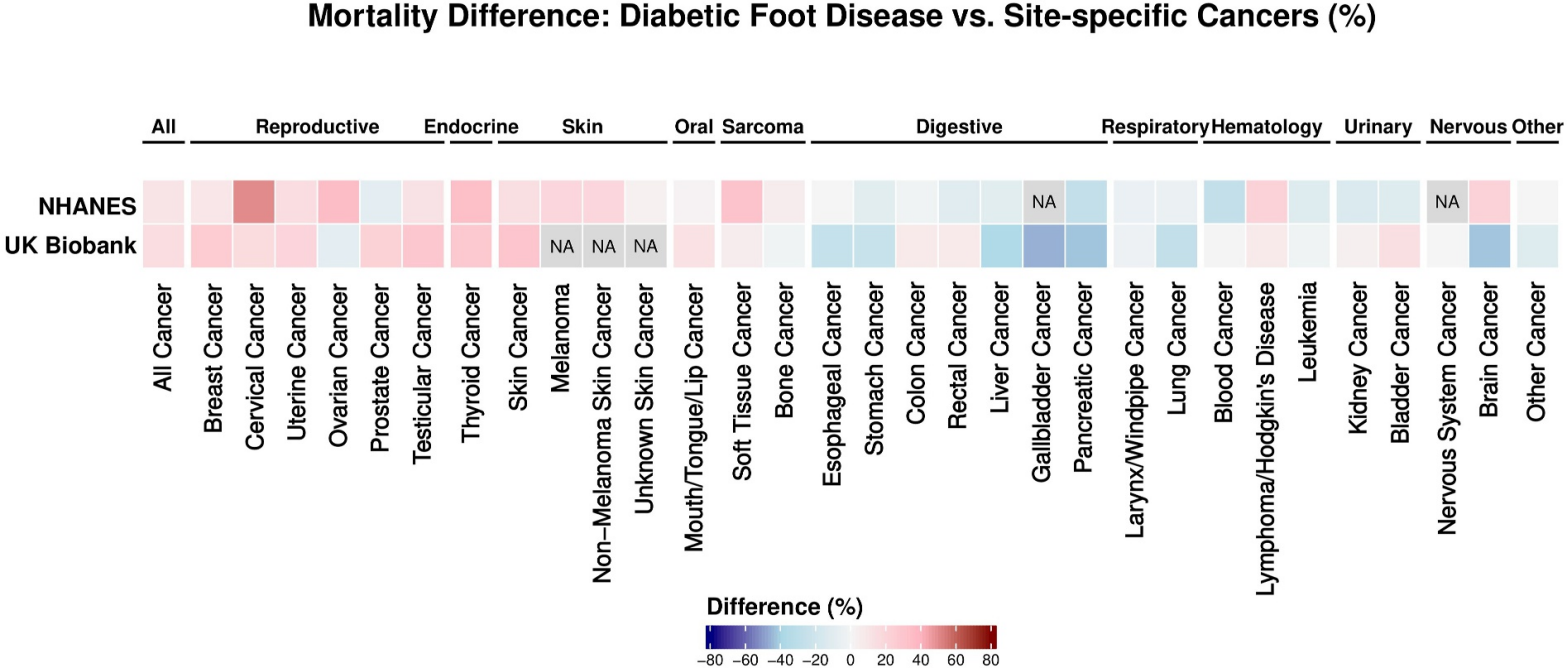
Mortality in patients with diabetic foot disease versus site‐specific cancers. Heatmap illustrating the differences in mortality between diabetic foot disease and site‐specific cancers in NHANES and UK Biobank. Each column represents a cancer type, and each row represents mortality differences in a specific cohort. Colours indicate the direction and magnitude of mortality differences (blue = lower mortality in diabetic foot disease compared to cancer, red = higher mortality in diabetic foot disease compared to cancer). Grey boxes indicate unavailable data.

**FIGURE 3:**
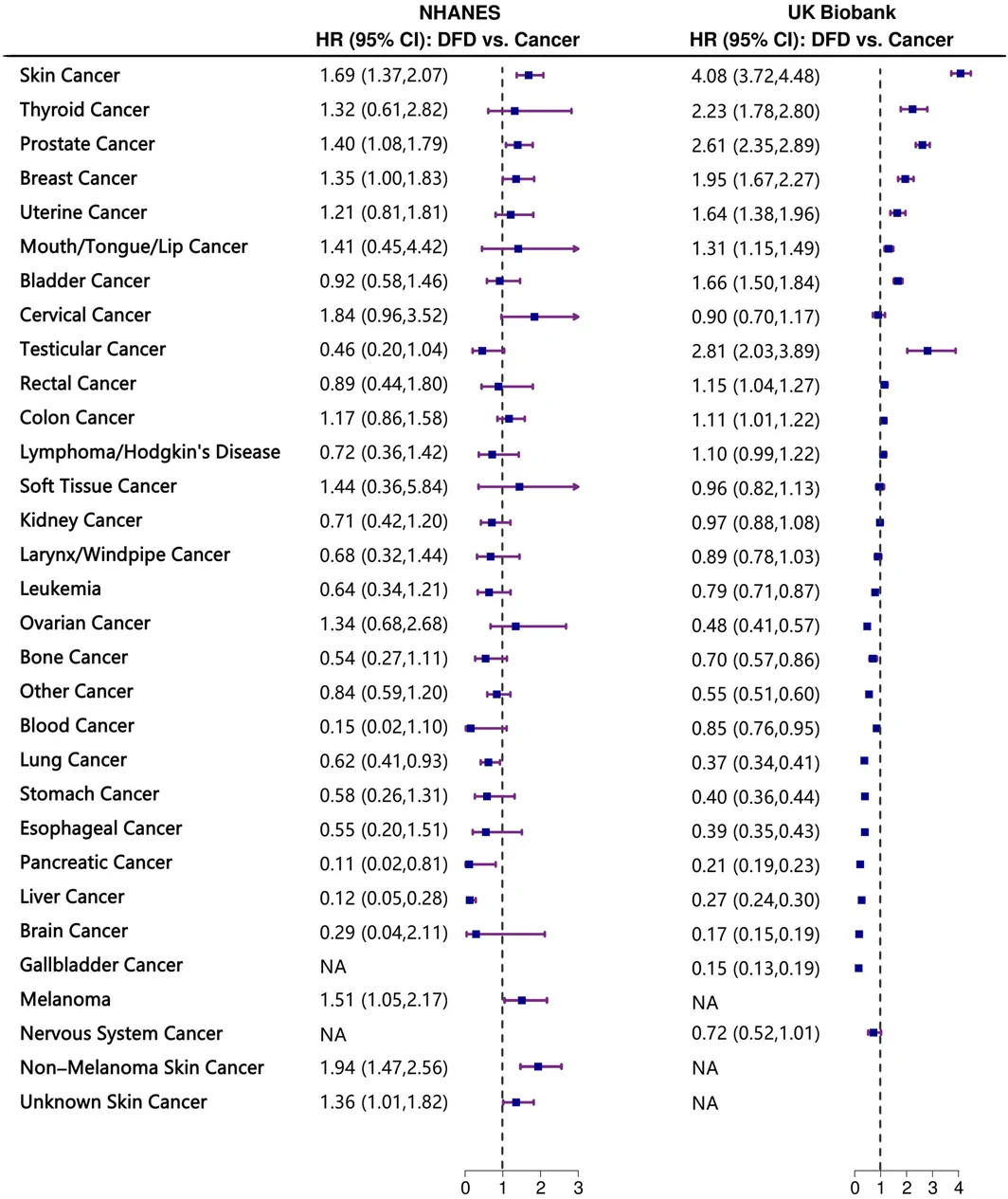
Mortality in patients with diabetic foot disease versus site‐specific cancers. Models were adjusted for age, sex, and body mass index. Squares represent HR estimates, and horizontal lines represent 95% CI.

The mortality rates for 31 site‐specific cancers were assessed in both the NHANES and UK Biobank cohorts (Supporting Information S1: Table S4). The differences in mortality between DFD and site‐specific cancers are shown as a heat map in Figure 2. Generally, DFD was associated with higher mortality compared with most site‐specific cancers, including melanoma and non‐melanoma, breast cancer, or prostate cancer, as reflected by the predominance of red shading across multiple cancer types in both cohorts. Notably, the risk was comparable to that of patients with cervical, colon, or kidney cancer, as well as those with lymphoma, soft tissue, laryngeal, and tracheal cancers. Conversely, the risk was lower than that of patients with lung, pancreatic, or liver cancers (Figure 3, Supporting Information S1: Table S5).

### 3.4 Sensitivity Analyses

Sensitivity analyses were conducted after excluding participants with heart failure or advanced chronic kidney disease (defined as an eGFR < 45 mL/min/1.73 m^2^). In both cohorts, the mortality risk was significantly higher in patients with DFD than in those with prostate or skin cancer. This was comparable to the risk observed in patients with cervical, colon, or kidney cancer, as well as lymphoma and soft tissue cancers, and lower than that in patients with liver, lung, or pancreatic cancer (Supporting Information S1: Tables S6–S9).

## 4 Discussion

Across three independent cohorts, our study demonstrated that patients with DFD had a higher mortality risk than those with other cancers did. For site‐specific cancers, the mortality risk of DFD was higher than that of skin, breast, and prostate cancers, comparable to that of colon cancer, lymphoma, and kidney cancer, and lower than that of lung, pancreatic, and liver cancers. These findings underscore the substantial burden of DFD. With the growing prevalence of diabetes worldwide [12, 13], the high morbidity and mortality of DFD calls for a re‐evaluation of its impact on public health.

Several guidelines and scientific statements from diabetes societies and the American Heart Association have recommended recognising DFD as a critical public health issue, asserting that its mortality risk is comparable to that of aggressive cancers [2, 3, 4]. Of note, the evidence underlying this recommendation originates from a commentary [8]. The DFD mortality data were obtained from previous multinational studies conducted in the United States, China, Japan, and several in Europe, between 2003 and 2019, with sample sizes ranging from 79 to 6992 [14, 15, 16, 17, 18, 19, 20, 21, 22, 23]. In contrast, the cancer mortality statistic was sourced solely from the American Cancer Society's 2019 U.S. report, which documented 606,880 deaths [24]. This commentary summarised 5‐year mortality rates for Charcot arthropathy (29.0%), diabetic foot ulcer (30.5%), minor amputation (46.2%), and major amputation (56.6%), which were comparable to the overall cancer mortality rate (31.0%), higher than those for breast (10%) and colon cancer (35%), and lower than that for lung cancer (80%). Significant heterogeneity in ethnicity, healthcare settings, and data collection periods across these studies introduces potential biases, thereby limiting the generalisability and reliability of the conclusions. To generate more rigorous evidence, we compared the mortality risk between DFD and cancer within the same cohort and validated the findings across three independent cohorts. Our results demonstrate that the mortality risk of DFD is not only higher than that of the overall cancer population but also higher or comparable to that of most site‐specific cancers, excluding lung, pancreatic, and liver cancers. As one report suggested, the costs of DFD nearly matched the $80.2 billion in direct costs for cancer in the U.S. [25, 26]. Given its high prevalence, severity, and economic burden, DFD should be recognized as a public health priority equivalent to cancer.

Although the incidence of cancer continues to rise, cancer mortality has been steadily declining [27, 28]. The age‐adjusted overall cancer mortality rate decreased by 33% between 1991 and 2021 [27]. This decline is attributed to public health interventions targeting risk factors and therapeutic advances. Advances in understanding key biological and pathophysiological pathways, including genetic, epigenetic, metabolic, and immune mechanisms, have yielded interventions such as molecular targeted therapy, endocrine therapy, and immunotherapy, substantially improving patient survival [29, 30]. In contrast, the situation for DFD is far from optimistic. Although longitudinal data on DFD mortality are limited, studies from both 2008 and 2023 report a consistent 5‐year mortality rate of approximately 50.4% [31, 32], Beyond known risk factors such as age, non‐Hispanic Black race, lower socioeconomic status, long duration of diabetes, and low BMI, cardiovascular disease, chronic kidney disease, and comorbid infections have been identified as significant risk factors for mortality in DFD [4, 14, 32, 33, 34, 35, 36, 37, 38]. Further analysis of primary causes of death in DFD patients revealed that cardiovascular and cerebrovascular diseases accounted for over 40% of deaths, confirming these diseases as the leading cause of mortality in this population (Supporting Information S1: Tables S10 and S11). Despite the mainstays of current treatments, including assessment of circulation, attention to infection, and survival outcomes for patients with DFD, they remain poor. Thus, critical knowledge gaps persist, encompassing both unidentified risk factors and incompletely elucidated pathophysiological mechanisms of the disease. To address these gaps, novel therapeutics and innovative strategies are urgently needed to overcome the current limitations of DFD management and improve patient survival.

The main strengths of our study include the large sample size and cross‐validation of the findings across three distinct international cohorts. The NHANES and UKB cohorts are nationally representative, whereas the CDR cohort provides data from the Chinese population, offering complementary perspectives. The consistent findings observed across these diverse cohorts significantly enhanced the robustness of our conclusions. However, this study has several limitations. Firstly, the CDR database lacked detailed information on cancer subtypes; however, the overall cancer risk estimates derived from it were highly consistent with those from the other two cohorts, which validates our primary findings. Secondly, the definition of cancer varied across cohorts, from self‐reported surveys in NHANES to ICD‐10 codes in UKB and physician‐diagnosed malignancy in CDR. Although efforts were made for harmonisation, these differences could introduce heterogeneity in cancer ascertainment. Similarly, while we adhered to the IWGDF 2023 criteria for DFD, the specific methods of assessment (e.g., self‐reported ulceration vs. objective ABI measurement) also varied, potentially affecting the precise classification of DFD cases. The follow‐up periods also differed substantially across cohorts (e.g., 4 years in CDR vs. 17 years in NHANES), which might influence the observed mortality rates, particularly for conditions with varying natural histories. These limitations highlight the complexity of comparing such diverse conditions and suggest avenues for future research with harmonised data collection protocols. Furthermore, for certain rare cancers, the limited number of cases may constrain statistical power, although this does not undermine the core conclusions regarding major cancer types and overall risk. Future studies with larger collaborative samples are warranted to further investigate these rare cancer subtypes.

## 5 Conclusion

Our comprehensive analysis, which involves multiple cohorts, presents the strong evidence showing that DFD carries a mortality burden that often surpasses, and at least rivals, that of most cancers. Specifically, our data indicate that the long‐term mortality risk associated with DFD is not only higher than that of all cancers combined but also exceeds the risks associated with common malignancies such as breast, prostate, and skin cancers. Furthermore, the risk is comparable to more serious conditions like colon, lymphoma, and kidney cancers, although it remains lower than that of aggressive cancers like lung, pancreatic, and liver cancers. This finding addresses a long‐standing gap in evidence within clinical guidelines and public health discussions. Public health strategies, clinical guidelines, and research funding bodies must recognise DFD not just as a complication of diabetes but also as a significant public health crisis comparable to prevalent cancers.

## Author Contributions

**Qinglian Zeng:** investigation, formal analysis, conceptualization, writing – original draft. **Ziwei Tang:** investigation, conceptualization, writing – review and editing. **Xiangjun Chen:** software, formal analysis, writing – original draft. **Linlin Zhan:** investigation, conceptualization, writing – review and editing. **Victoria Balasubramanian:** writing – review and editing. **Zhihao Chen, Manman Du, Qi Zhang, Fei Lian, Xiaoru Zhang, Qing Liang, Wenrui Zhao, Xun Li, Mengyao Zhang:** investigation. **Qifu Li:** conceptualization, supervision. **Shumin Yang:** conceptualization, writing – review and editing. **Jinbo Hu:** conceptualization, investigation, data curation. **Qingfeng Cheng:** resources, project administration, supervision. **Hang Shen:** conceptualization, supervision, writing – review and editing.

## Funding

This work was supported by the National Natural Science Foundation of China (Grant 82270878, Jinbo Hu; Grant 82501003, Xiangjun Chen), Chongqing Outstanding Youth Science Fund project (Grant CSTB2023NSCQ‐JQX0028, Jinbo Hu), the Science and Technology Research Programme of Chongqing Municipal Education Commission (Grant KJZD‐K202500409, Jinbo Hu), Programme for Youth Innovation in Future Medicine, Chongqing Medical University (Grant W0187, Ziwei Tang), Foundation of Sichuan‐Chongqing Joint Key Laboratory of Metabolic Vascular Diseases, Chongqing Key Laboratory of Translational Medicine in Major Metabolic Diseases (Grant DXXXGJB‐CYGY‐KFKT‐202501, Ziwei Tang), the Key Project of Special Technological Innovation and Application Development in Chongqing (Grant CSTB2024TIAD‐KPX0039, Qifu Li), and Joint Medical Research Project for Technological Innovation in Chongqing Health Commission & Chongqing Science and Technology Commission (Grant 2025GGXM004, Qifu Li).

## Conflicts of Interest

The authors declare no conflicts of interest.

## Reproducible Research Statement

Study protocol and statistical code: Available from Dr. Jinbo Hu, PhD (e‐mail: hujinbo@cqmu.edu.cn).

## Supporting information


Supporting Information S1

## Data Availability

The data that support the findings of this study are available from the corresponding author upon reasonable request.
