# Supplementary material for: Long‐Term Mortality in Diabetic Foot Disease is Higher Than That of Overall Cancer: Results From Three International Cohorts
Source: Diabetes Metab Res Rev. 2026 Sep 8;42(6):e70221. doi: 10.1002/dmrr.70221 (PMC13552382; doi:10.1002/dmrr.70221)
Supplement: Supplementary file 1 [file DMRR-42-e70221-s001.doc]

**Supplementary Table 1. ICD-10 code list for malignant neoplasms.**

| **ICD10 Code** | **Description** | **Categories** |
| --- | --- | --- |
| C01 | Malignant neoplasm of base of tongue | Mouth/Tongue/Lip Cancer |
| C02 | Malignant neoplasm of other and unspecified parts of tongue | Mouth/Tongue/Lip Cancer |
| C03 | Malignant neoplasm of gum | Mouth/Tongue/Lip Cancer |
| C04 | Malignant neoplasm of floor of mouth | Mouth/Tongue/Lip Cancer |
| C05 | Malignant neoplasm of palate | Mouth/Tongue/Lip Cancer |
| C06 | Malignant neoplasm of other and unspecified parts of mouth | Mouth/Tongue/Lip Cancer |
| C07 | Malignant neoplasm of parotid gland | Mouth/Tongue/Lip Cancer |
| C08 | Malignant neoplasm of other and unspecified major salivary glands | Mouth/Tongue/Lip Cancer |
| C09 | Malignant neoplasm of tonsil | Mouth/Tongue/Lip Cancer |
| C10 | Malignant neoplasm of oropharynx | Larynx/Windpipe Cancer |
| C11 | Malignant neoplasm of nasopharynx | Larynx/Windpipe Cancer |
| C12 | Malignant neoplasm of pyriform sinus | Larynx/Windpipe Cancer |
| C13 | Malignant neoplasm of hypopharynx | Larynx/Windpipe Cancer |
| C14 | Malignant neoplasm of other and ill-defined sites in the lip, oral cavity and pharynx | Larynx/Windpipe Cancer |
| C15 | Malignant neoplasm of oesophagus | Esophageal Cancer |
| C16 | Malignant neoplasm of stomach | Stomach Cancer |
| C17 | Malignant neoplasm of small intestine | Other cancer |
| C18 | Malignant neoplasm of colon | Colon Cancer |
| C19 | Malignant neoplasm of rectosigmoid junction | Rectal Cancer |
| C20 | Malignant neoplasm of rectum | Rectal Cancer |
| C21 | Malignant neoplasm of anus and anal canal | Rectal Cancer |
| C22 | Malignant neoplasm of liver and intrahepatic bile ducts | Liver Cancer |
| C23 | Malignant neoplasm of gallbladder | Gallbladder Cancer |
| C24 | Malignant neoplasm of other and unspecified parts of biliary tract | Other cancer |
| C25 | Malignant neoplasm of pancreas | Pancreatic Cancer |
| C26 | Malignant neoplasm of other and ill-defined digestive organs | Other cancer |
| C30 | Malignant neoplasm of nasal cavity and middle ear | Other cancer |
| C31 | Malignant neoplasm of accessory sinuses | Other cancer |
| C32 | Malignant neoplasm of larynx | Larynx/Windpipe Cancer |
| C33 | Malignant neoplasm of trachea | Lung Cancer |
| C34 | Malignant neoplasm of bronchus and lung | Lung Cancer |
| C37 | Malignant neoplasm of thymus | Other cancer |
| C39 | Malignant neoplasm of other and ill-defined sites in the respiratory system and intrathoracic organs | Other cancer |
| C40 | Malignant neoplasm of bone and articular cartilage of limbs | Bone Cancer |
| C41 | Malignant neoplasm of bone and articular cartilage of other and unspecified sites | Bone Cancer |
| C43 | Malignant melanoma of skin | Skin Cancer |
| C44 | Other malignant neoplasms of skin | Skin Cancer |
| C45 | Mesothelioma | Other cancer |
| C46 | Kaposi's sarcoma | Soft Tissue Cancer |
| C47 | Malignant neoplasm of peripheral nerves and autonomic nervous system | Nervous System Cancer |
| C48 | Malignant neoplasm of retroperitoneum and peritoneum | Other cancer |
| C49 | Malignant neoplasm of other connective and soft tissue | Soft Tissue Cancer |
| C50 | Malignant neoplasm of breast | Breast Cancer |
| C51 | Malignant neoplasm of vulva | Other cancer |
| C52 | Malignant neoplasm of vagina | Other cancer |
| C53 | Malignant neoplasm of cervix uteri | Cervical Cancer |
| C54 | Malignant neoplasm of corpus uteri | Uterine Cancer |
| C55 | Malignant neoplasm of uterus, part unspecified | Uterine Cancer |
| C56 | Malignant neoplasm of ovary | Ovarian Cancer |
| C57 | Malignant neoplasm of other and unspecified female genital organs | Other cancer |
| C58 | Malignant neoplasm of placenta | Other cancer |
| C60 | Malignant neoplasm of penis | Other cancer |
| C61 | Malignant neoplasm of prostate | Prostate Cancer |
| C62 | Malignant neoplasm of testis | Testicular Cancer |
| C63 | Malignant neoplasm of other and unspecified male genital organs | Other cancer |
| C64 | Malignant neoplasm of kidney, except renal pelvis | Kidney Cancer |
| C65 | Malignant neoplasm of renal pelvis | Kidney Cancer |
| C66 | Malignant neoplasm of ureter | Other cancer |
| C67 | Malignant neoplasm of bladder | Bladder Cancer |
| C68 | Malignant neoplasm of other and unspecified urinary organs | Other cancer |
| C69 | Malignant neoplasm of eye and adnexa | Other cancer |
| C70 | Malignant neoplasm of meninges | Other cancer |
| C71 | Malignant neoplasm of brain | Brain Cancer |
| C72 | Malignant neoplasm of spinal cord, cranial nerves and other parts of central nervous system | Nervous System Cancer |
| C73 | Malignant neoplasm of thyroid gland | Thyroid Cancer |
| C74 | Malignant neoplasm of adrenal gland | Other cancer |
| C75 | Malignant neoplasm of other endocrine glands and related structures | Other cancer |
| C76 | Malignant neoplasm of other and ill-defined sites | Other cancer |
| C80 | Malignant neoplasm without specification of site | Other cancer |
| C81 | Hodgkin's disease | Lymphoma/Hodgkin's Disease |
| C82 | Follicular [nodular] non-Hodgkin's lymphoma | Lymphoma/Hodgkin's Disease |
| C83 | Diffuse non-Hodgkin's lymphoma | Lymphoma/Hodgkin's Disease |
| C84 | Peripheral and cutaneous T-cell lymphomas | Lymphoma/Hodgkin's Disease |
| C85 | Other and unspecified types of non-Hodgkin's lymphoma | Lymphoma/Hodgkin's Disease |
| C86 | Other specified types of T/NK-cell lymphoma | Lymphoma/Hodgkin's Disease |
| C88 | Malignant immunoproliferative diseases | Blood Cancer |
| C90 | Multiple myeloma and malignant plasma cell neoplasms | Blood Cancer |
| C91 | Lymphoid leukaemia | Leukemia |
| C92 | Myeloid leukaemia | Leukemia |
| C93 | Monocytic leukaemia | Leukemia |
| C94 | Other leukaemias of specified cell type | Leukemia |
| C95 | Leukaemia of unspecified cell type | Leukemia |
| C96 | Other and unspecified malignant neoplasms of lymphoid, haematopoietic and related tissue | Leukemia |
| C97 | Malignant neoplasms of independent (primary)multiple sites | Other cancer |

**Supplementary Table 2. Overall, 1-year, 5-year, and 10-year mortality in participants with cancer, diabetes, and diabetic foot disease across three cohorts.**

|  | **Mortality** | | |
| --- | --- | --- | --- |
|  | **Cancer** | **Diabetes** | **Diabetic Foot Disease** |
| **NHANES** |  |  |  |
| Follow-up time | 14.0(6.1,17.2) | 16.5(12.9,18.3) | 11.6(5.5,16.3) |
| Total | 61.0% | 41.4% | 71.7% |
| 1 year | 4.2% | 0.3% | 3.7% |
| 5 year | 23.5% | 6.6% | 23.1% |
| 10 year | 41.3% | 17.5% | 45.8% |
| **UKB** |  |  |  |
| Follow-up time | 13.4(12.4,14.2) | 13.0(5.5,18.8) | 18.8(11.5,26.1) |
| Total | 25.5% | 11.4% | 40.6% |
| 1 year | 9.7% | 1.4% | 12.5% |
| 5 year | 18.4% | 3.3% | 25.5% |
| 10 year | 22.2% | 5.3% | 33.2% |
| **CDR** |  |  |  |
| Follow-up time | 4.0 (3.0, 5.0) | 4.0 (3.0, 5.0) | 4.4 (3.0, 6.0) |
| Total | 8.6% | 4.4% | 34.4% |
| 1 year | 2.6% | 0.8% | 8.3% |
| 5 year | 8.6% | 4.3% | 26.7% |

Abbreviations: NHANES, National Health and Nutrition Examination Survey; UKB, UK Biobank; CDR, Chongqing Diabetes Registry.

**Supplementary Table 3. Mortality in patients with diabetic foot disease versus cancer**

|  | **Diabetic foot Disease** | |  | **Cancer** | |  | **Hazard Ratio (95% CI)** |
| --- | --- | --- | --- | --- | --- | --- | --- |
|  | Events | Total |  | Events | Total |  |  |
| **NHANES** | 410 | 572 |  | 744 | 1219 |  | 1.37(1.18, 1.59) |
| **UKB** | 687 | 1691 |  | 25649 | 100736 |  | 1.46(1.35, 1.58) |
| **CDR** | 233 | 678 |  | 13 | 152 |  | 5.99(1.88, 19.12) |

Abbreviations: NHANES, National Health and Nutrition Examination Survey; UKB, UK Biobank; CDR, Chongqing Diabetes Registry; CI, confidence interval.

Supplementary Table 4. Mortality at 1, 5, and 10 years for site-specific cancers in the NHANES and UK biobank.

|  | NHANES | | | |  | UK Biobank | | | |
| --- | --- | --- | --- | --- | --- | --- | --- | --- | --- |
|  | overall | 1 year | 5 year | 10 year |  | overall | 1 year | 5 year | 10 year |
| All Cancer | 61.0% | 4.2% | 23.5% | 41.3% |  | 25.5% | 9.7% | 18.4% | 22.2% |
| Mouth/Tongue/Lip Cancer | 70.0% | 10.0% | 50.0% | 50.0% |  | 28.2% | 7.2% | 17.5% | 22.4% |
| Larynx/Windpipe Cancer | 77.8% | 22.2% | 33.3% | 55.6% |  | 44.5% | 16.8% | 30.8% | 38.0% |
| Esophageal Cancer | 71.4% | 42.9% | 42.9% | 57.1% |  | 67.0% | 39.9% | 60.9% | 64.7% |
| Stomach Cancer | 81.8% | 0.0% | 36.4% | 45.5% |  | 65.9% | 40.1% | 60.7% | 63.8% |
| Colon Cancer | 74.5% | 8.2% | 36.7% | 62.2% |  | 33.5% | 12.9% | 26.7% | 30.9% |
| Rectal Cancer | 83.3% | 0.0% | 33.3% | 66.7% |  | 33.1% | 11.2% | 24.8% | 29.5% |
| Liver Cancer | 81.8% | 45.5% | 72.7% | 72.7% |  | 78.3% | 53.6% | 74.8% | 77.7% |
| Gallbladder Cancer | / | / | / | / |  | 87.5% | 68.5% | 85.3% | 87.0% |
| Pancreatic Cancer | 100.0% | 0.0% | 100.0% | 100.0% |  | 83.8% | 64.0% | 82.2% | 83.1% |
| Lung Cancer | 77.3% | 15.9% | 52.3% | 70.5% |  | 69.2% | 45.8% | 65.3% | 67.9% |
| Skin Cancer | 57.0% | 2.0% | 16.5% | 32.1% |  | 10.5% | 1.4% | 5.5% | 8.4% |
| Melanoma | 52.6% | 1.3% | 18.4% | 27.6% |  | / | / | / | / |
| Non-Melanoma Skin Cancer | 52.5% | 2.2% | 13.3% | 29.3% |  | / | / | / | / |
| Unknown Skin Cancer | 68.9% | 1.9% | 21.7% | 42.5% |  | / | / | / | / |
| Soft Tissue Cancer | 40.0% | 0.0% | 0.0% | 40.0% |  | 35.7% | 16.6% | 29.3% | 33.9% |
| Breast Cancer | 62.1% | 2.7% | 20.3% | 41.2% |  | 14.7% | 1.7% | 6.4% | 9.9% |
| Cervical Cancer | 21.7% | 0.0% | 3.6% | 12.0% |  | 24.5% | 8.6% | 18.5% | 21.6% |
| Uterine Cancer | 56.3% | 1.6% | 15.6% | 28.1% |  | 20.5% | 5.1% | 14.9% | 17.8% |
| Ovarian Cancer | 36.7% | 0.0% | 16.7% | 23.3% |  | 49.8% | 15.8% | 39.6% | 46.9% |
| Prostate Cancer | 80.7% | 4.8% | 33.8% | 59.4% |  | 18.1% | 4.0% | 11.5% | 15.6% |
| Testicular Cancer | 60.0% | 0.0% | 30.0% | 30.0% |  | 12.1% | 2.2% | 5.0% | 6.3% |
| Kidney Cancer | 86.7% | 10.0% | 43.3% | 73.3% |  | 37.2% | 14.8% | 28.5% | 33.5% |
| Bladder Cancer | 84.8% | 9.1% | 33.3% | 66.7% |  | 26.7% | 8.3% | 18.0% | 22.7% |
| Nervous System Cancer | / | / | / | / |  | 40.4% | 18.1% | 28.7% | 36.2% |
| Brain Cancer | 50.0% | 0.0% | 25.0% | 25.0% |  | 83.6% | 56.5% | 79.6% | 81.4% |
| Thyroid Cancer | 38.1% | 9.5% | 14.3% | 23.8% |  | 13.3% | 4.1% | 8.4% | 11.2% |
| Blood Cancer | 100.0% | 0.0% | 100.0% | 100.0% |  | 41.4% | 12.4% | 28.5% | 37.9% |
| Lymphoma/Hodgkin's Disease | 50.0% | 18.2% | 40.9% | 45.5% |  | 32.8% | 11.8% | 22.0% | 27.7% |
| Leukemia | 84.6% | 7.7% | 53.8% | 61.5% |  | 43.6% | 20.2% | 34.8% | 40.0% |
| Bone Cancer | 66.7% | 5.6% | 44.4% | 55.6% |  | 42.7% | 18.4% | 34.3% | 38.6% |
| Other Cancer | 71.4% | 7.9% | 28.6% | 46.0% |  | 53.9% | 31.8% | 49.0% | 52.2% |

**Supplementary Table 5. Mortality in patients with diabetic foot disease versus site-specific cancers.**

|  | **NHANES** | | | |  | **UK Biobank** | | | |
| --- | --- | --- | --- | --- | --- | --- | --- | --- | --- |
|  | No. of Participants | No. of Death | HR (95% CI)for DFD vs. Cancer | *P* value |  | No. of Participants | No. of Death | HR (95% CI)for DFD vs. Cancer | *P* value |
| Diabetic foot | 572 | 410 |  |  |  | 1691 | 687 |  |  |
| Skin Cancer | 358 | 204 | 1.69 (1.37,2.07) | <0.001 |  | 29645 | 3104 | 4.08 (3.72, 4.48) | <0.001 |
| Thyroid Cancer | 21 | 8 | 1.32 (0.61,2.82) | 0.481 |  | 769 | 102 | 2.23 (1.78, 2.80) | <0.001 |
| Prostate Cancer | 207 | 167 | 1.40 (1.08,1.79) | 0.010 |  | 15385 | 2791 | 2.61 (2.35, 2.89) | <0.001 |
| Breast Cancer | 182 | 113 | 1.35 (1.00,1.83) | 0.050 |  | 18948 | 2786 | 1.95 (1.67, 2.27) | <0.001 |
| Uterine Cancer | 64 | 36 | 1.21 (0.81,1.81) | 0.353 |  | 2741 | 562 | 1.64 (1.38, 1.96) | <0.001 |
| Mouth/Tongue/Lip Cancer | 10 | 7 | 1.41 (0.45,4.42) | 0.557 |  | 1721 | 486 | 1.31 (1.15, 1.49) | <0.001 |
| Bladder Cancer | 33 | 28 | 0.92 (0.58,1.46) | 0.717 |  | 4751 | 1270 | 1.66 (1.50, 1.84) | <0.001 |
| Cervical Cancer | 83 | 18 | 1.84 (0.96,3.52) | 0.065 |  | 440 | 108 | 0.90 (0.70, 1.17) | 0.433 |
| Testicular Cancer | 10 | 6 | 0.46 (0.20,1.04) | 0.061 |  | 363 | 44 | 2.81 (2.03, 3.89) | <0.001 |
| Rectal Cancer | 12 | 10 | 0.89 (0.44,1.80) | 0.736 |  | 4487 | 1485 | 1.15 (1.04, 1.27) | 0.008 |
| Colon Cancer | 98 | 73 | 1.17 (0.86,1.58) | 0.328 |  | 7125 | 2390 | 1.11 (1.01, 1.22) | 0.024 |
| Lymphoma/Hodgkin's Disease | 22 | 11 | 0.72 (0.36,1.42) | 0.344 |  | 4326 | 1417 | 1.10 (0.99, 1.22) | 0.068 |
| Soft Tissue Cancer | 5 | 2 | 1.44 (0.36,5.84) | 0.609 |  | 672 | 240 | 0.96 (0.82, 1.13) | 0.653 |
| Kidney Cancer | 30 | 26 | 0.71 (0.42,1.20) | 0.203 |  | 2615 | 972 | 0.97 (0.88, 1.08) | 0.614 |
| Larynx/Windpipe Cancer | 9 | 7 | 0.68 (0.32,1.44) | 0.312 |  | 804 | 358 | 0.89 (0.78, 1.03) | 0.116 |
| Leukemia | 13 | 11 | 0.64 (0.34,1.21) | 0.166 |  | 2670 | 1165 | 0.79 (0.71, 0.87) | <0.001 |
| Ovarian Cancer | 30 | 11 | 1.34 (0.68,2.68) | 0.400 |  | 1987 | 990 | 0.48 (0.41, 0.57) | <0.001 |
| Bone Cancer | 18 | 12 | 0.54 (0.27,1.11) | 0.094 |  | 274 | 117 | 0.70 (0.57, 0.86) | 0.001 |
| Other Cancer | 63 | 45 | 0.84 (0.59,1.20) | 0.341 |  | 8747 | 4713 | 0.55 (0.51, 0.60) | <0.001 |
| Blood Cancer | 1 | 1 | 0.15 (0.02,1.10) | 0.062 |  | 1935 | 801 | 0.85 (0.76, 0.95) | 0.004 |
| Lung Cancer | 44 | 34 | 0.62 (0.41,0.93) | 0.019 |  | 6365 | 4403 | 0.37 (0.34, 0.41) | <0.001 |
| Stomach Cancer | 11 | 9 | 0.58 (0.26,1.31) | 0.192 |  | 1474 | 971 | 0.40 (0.36, 0.44) | <0.001 |
| Esophageal Cancer | 7 | 5 | 0.55 (0.20,1.51) | 0.246 |  | 1942 | 1302 | 0.39 (0.35, 0.43) | <0.001 |
| Pancreatic Cancer | 1 | 1 | 0.11 (0.02,0.81) | 0.031 |  | 2011 | 1685 | 0.21 (0.19, 0.23) | <0.001 |
| Liver Cancer | 11 | 9 | 0.12 (0.05,0.28) | <0.001 |  | 1160 | 908 | 0.27 (0.24, 0.30) | <0.001 |
| Brain Cancer | 4 | 2 | 0.29 (0.04,2.11) | 0.222 |  | 1198 | 1001 | 0.17 (0.15, 0.19) | <0.001 |
| Gallbladder Cancer | 0 | 0 | / | / |  | 184 | 161 | 0.15 (0.13, 0.19) | <0.001 |
| Melanoma | 76 | 40 | 1.51 (1.05,2.17) | 0.028 |  | / | / | / | / |
| Nervous System Cancer | 0 | 0 | / | / |  | 94 | 38 | 0.72 (0.52, 1.01) | 0.060 |
| Non-Melanoma Skin Cancer | 181 | 95 | 1.94 (1.47,2.56) | <0.001 |  | / | / | / | / |
| Unknown Skin Cancer | 106 | 73 | 1.36 (1.01,1.82) | 0.043 |  | / | / | / | / |

Models were adjusted for age ,sex, and body mass index. Squares represent HR estimates, and horizontal lines represent 95%CIs.

**Supplementary Table 6. Mortality in patients with diabetic foot disease versus site-specific cancers in the NHANES cohort after excluding participants with heart failure**

|  | **No. of Participants** | **No. of Death** | **HR (95% CI): Diabetic Foot vs. Cancer** | ***P value*** |
| --- | --- | --- | --- | --- |
| Diabetic foot | 495 | 338 |  |  |
| Skin Cancer | 336 | 188 | 1.54 (1.24,1.92) | <0.001 |
| Thyroid Cancer | 19 | 6 | 1.28 (0.52,3.18) | 0.591 |
| Prostate Cancer | 189 | 151 | 1.32 (1.01,1.72) | 0.042 |
| Breast Cancer | 170 | 101 | 1.29 (0.94,1.79) | 0.120 |
| Uterine Cancer | 62 | 34 | 1.12 (0.73,1.70) | 0.611 |
| Mouth/Tongue/Lip Cancer | 8 | 5 | 1.96 (0.48,7.91) | 0.347 |
| Bladder Cancer | 32 | 28 | 0.82 (0.51,1.30) | 0.396 |
| Cervical Cancer | 81 | 17 | 1.75 (0.88,3.46) | 0.110 |
| Testicular Cancer | 10 | 6 | 0.40 (0.18,0.92) | 0.032 |
| Rectal Cancer | 11 | 9 | 0.81 (0.40,1.64) | 0.554 |
| Colon Cancer | 91 | 66 | 1.15 (0.83,1.59) | 0.404 |
| Lymphoma/Hodgkin's Disease | 20 | 9 | 0.83 (0.38,1.79) | 0.631 |
| Soft Tissue Cancer | 4 | 1 | 2.27 (0.32,16.37) | 0.415 |
| Kidney Cancer | 27 | 23 | 0.65 (0.38,1.13) | 0.125 |
| Larynx/Windpipe Cancer | 6 | 4 | 0.97 (0.36,2.62) | 0.946 |
| Leukemia | 12 | 10 | 0.57 (0.30,1.09) | 0.090 |
| Ovarian Cancer | 29 | 10 | 1.19 (0.59,2.38) | 0.625 |
| Bone Cancer | 14 | 8 | 0.62 (0.27,1.41) | 0.251 |
| Other Cancer | 56 | 38 | 0.78 (0.53,1.16) | 0.218 |
| Blood Cancer | 1 | 1 | 0.12 (0.02,0.87) | 0.036 |
| Lung Cancer | 41 | 31 | 0.58 (0.38,0.87) | 0.009 |
| Stomach Cancer | 10 | 8 | 0.53 (0.23,1.19) | 0.122 |
| Esophageal Cancer | 3 | 1 | 1.06 (0.15,7.66) | 0.953 |
| Pancreatic Cancer | 1 | 1 | 0.10 (0.01,0.71) | 0.022 |
| Liver Cancer | 11 | 9 | 0.10 (0.05,0.24) | <0.001 |
| Brain Cancer | 4 | 2 | 0.26 (0.04,1.88) | 0.182 |
| Gallbladder Cancer | 0 | 0 | / | / |
| Melanoma | 73 | 38 | 1.39 (0.95,2.03) | 0.092 |
| Nervous System Cancer | 0 | 0 | / | / |
| Non-Melanoma Skin Cancer | 170 | 87 | 1.79 (1.33,2.39) | <0.001 |
| Unknown Skin Cancer | 97 | 66 | 1.24 (0.91,1.69) | 0.166 |

Models were adjusted for age ,sex, and body mass index. Squares represent HR estimates, and horizontal lines represent 95% CIs.

**^Supplementary Table 7. Mortality in patients with diabetic foot disease versus site-specific cancers in the NHANES cohort after excluding participants with estimated glomerular filtration rate less than 45 ml/min/1.73m2^**

|  | **No. of Participants** | **No. of Death** | **HR (95% CI): Diabetic Foot vs. Cancer** | ***P value*** |
| --- | --- | --- | --- | --- |
| Diabetic foot | 497 | 347 |  |  |
| Skin Cancer | 339 | 186 | 1.63 (1.31,2.03) | <0.001 |
| Thyroid Cancer | 20 | 7 | 1.30 (0.57,2.97) | 0.532 |
| Prostate Cancer | 198 | 159 | 1.32 (1.01,1.71) | 0.039 |
| Breast Cancer | 174 | 106 | 1.30 (0.95,1.80) | 0.107 |
| Uterine Cancer | 59 | 32 | 1.22 (0.79,1.89) | 0.362 |
| Mouth/Tongue/Lip Cancer | 10 | 7 | 1.34 (0.43,4.21) | 0.619 |
| Bladder Cancer | 27 | 22 | 0.94 (0.56,1.57) | 0.801 |
| Cervical Cancer | 81 | 16 | 2.02 (0.99,4.12) | 0.055 |
| Testicular Cancer | 10 | 6 | 0.41 (0.18,0.94) | 0.036 |
| Rectal Cancer | 12 | 10 | 0.82 (0.40,1.66) | 0.573 |
| Colon Cancer | 91 | 68 | 1.08 (0.79,1.49) | 0.620 |
| Lymphoma/Hodgkin's Disease | 22 | 11 | 0.67 (0.34,1.33) | 0.250 |
| Soft Tissue Cancer | 5 | 2 | 1.28 (0.32,5.21) | 0.728 |
| Kidney Cancer | 18 | 16 | 0.70 (0.34,1.43) | 0.326 |
| Larynx/Windpipe Cancer | 9 | 7 | 0.63 (0.29,1.35) | 0.233 |
| Leukemia | 13 | 11 | 0.60 (0.31,1.14) | 0.115 |
| Ovarian Cancer | 29 | 11 | 1.09 (0.54,2.20) | 0.800 |
| Bone Cancer | 17 | 11 | 0.54 (0.25,1.17) | 0.117 |
| Other Cancer | 60 | 42 | 0.75 (0.52,1.10) | 0.144 |
| Blood Cancer | 1 | 1 | 0.14 (0.02,1.04) | 0.054 |
| Lung Cancer | 43 | 33 | 0.60 (0.40,0.91) | 0.016 |
| Stomach Cancer | 11 | 9 | 0.54 (0.24,1.22) | 0.139 |
| Esophageal Cancer | 6 | 4 | 0.51 (0.19,1.39) | 0.186 |
| Pancreatic Cancer | 0 | 0 | / | / |
| Liver Cancer | 10 | 8 | 0.11 (0.05,0.26) | <0.001 |
| Brain Cancer | 4 | 2 | 0.26 (0.04,1.90) | 0.185 |
| Gallbladder Cancer | 0 | 0 | / | / |
| Melanoma | 73 | 37 | 1.49 (1.01,2.19) | 0.043 |
| Nervous System Cancer | 0 | 0 | / | / |
| Non-Melanoma Skin Cancer | 173 | 88 | 1.85 (1.39,2.47) | <0.001 |
| Unknown Skin Cancer | 97 | 64 | 1.34 (0.98,1.83) | 0.069 |

Models were adjusted for age ,sex, and body mass index. Squares represent HR estimates, and horizontal lines represent 95%CIs.

**Supplementary Table 8. Mortality in patients with diabetic foot disease versus site-specific cancers in the UK Biobank cohort after excluding participants with heart failure**

|  | **No. of Participants** | **No. of Death** | **HR (95% CI):**  **Diabetic Foot vs. Cancer** | ***P value*** |
| --- | --- | --- | --- | --- |
| Diabetic foot | 1346 | 486 |  |  |
| Skin Cancer | 29431 | 3038 | 3.54 (3.19, 3.93) | <0.001 |
| Thyroid Cancer | 766 | 101 | 1.83 (1.44, 2.32) | <0.001 |
| Prostate Cancer | 15240 | 2722 | 2.35 (2.09, 2.649) | <0.001 |
| Breast Cancer | 18905 | 2768 | 1.47 (1.23, 1.75) | <0.001 |
| Uterine Cancer | 2734 | 559 | 1.26 (1.02, 1.55) | 0.033 |
| Mouth/Tongue/Lip Cancer | 1704 | 474 | 1.14 (0.99, 1.32) | 0.067 |
| Bladder Cancer | 4689 | 1234 | 1.44 (1.28, 1.62) | <0.001 |
| Cervical Cancer | 436 | 106 | 0.69 (0.52, 0.91) | 0.009 |
| Testicular Cancer | 358 | 42 | 2.62 (1.86, 3.68) | <0.001 |
| Rectal Cancer | 4463 | 1468 | 0.97 (0.87, 1.09) | 0.634 |
| Colon Cancer | 7073 | 2364 | 0.94 (0.85, 1.05) | 0.250 |
| Lymphoma/Hodgkin's Disease | 4273 | 1387 | 0.94 (0.84, 1.05) | 0.291 |
| Soft Tissue Cancer | 661 | 236 | 0.82 (0.69, 0.97) | 0.020 |
| Kidney Cancer | 2586 | 953 | 0.83 (0.74, 0.94) | 0.002 |
| Larynx/Windpipe Cancer | 791 | 349 | 0.79 (0.68, 0.92) | 0.002 |
| Leukemia | 2638 | 1143 | 0.68 (0.61, 0.76) | <0.001 |
| Ovarian Cancer | 1984 | 988 | 0.37 (0.30, 0.45) | <0.001 |
| Bone Cancer | 270 | 113 | 0.59 (0.48, 0.74) | <0.001 |
| Other Cancer | 8664 | 4650 | 0.47 (0.43, 0.52) | <0.001 |
| Blood Cancer | 1916 | 784 | 0.73 (0.64, 0.82) | <0.001 |
| Lung Cancer | 6279 | 4331 | 0.32 (0.29, 0.35) | <0.001 |
| Stomach Cancer | 1459 | 958 | 0.33 (0.50, 0.38) | <0.001 |
| Esophageal Cancer | 1919 | 1281 | 0.33 (0.30, 0.37) | <0.001 |
| Pancreatic Cancer | 1996 | 1670 | 0.18 (0.16, 0.20) | <0.001 |
| Liver Cancer | 1141 | 890 | 0.23 (0.20, 0.26) | <0.001 |
| Brain Cancer | 1191 | 994 | 0.14 (0.13, 0.16) | <0.001 |
| Gallbladder Cancer | 183 | 160 | 0.12 (0.10, 0.14) | <0.001 |
| Melanoma | / | / | / | / |
| Nervous System Cancer | 93 | 37 | 0.62 (0.44, 0.88) | 0.007 |
| Non-Melanoma Skin Cancer | / | / | / | / |
| Unknown Skin Cancer | / | / | / | / |

Models were adjusted for age ,sex, and body mass index. Squares represent HR estimates, and horizontal lines represent 95%CIs.

**^Supplementary Table 9. Mortality in patients with diabetic foot disease versus site-specific cancers in the UK Biobank cohort after excluding participants with estimated glomerular filtration rate less than 45 ml/min/1.73m2^**

|  | **No. of Participants** | **No. of Death** | **HR (95% CI): Diabetic Foot vs. Cancer** | ***P value*** |
| --- | --- | --- | --- | --- |
| Diabetic foot | 1607 | 618 |  |  |
| Skin Cancer | 29445 | 3028 | 3.91 (3.55, 4.30) | <0.001 |
| Thyroid Cancer | 766 | 101 | 2.08 (1.65, 2.62) | <0.001 |
| Prostate Cancer | 15306 | 2744 | 2.51 (2.26, 2.80) | <0.001 |
| Breast Cancer | 18868 | 2760 | 1.79 (1.52, 2.10) | <0.001 |
| Uterine Cancer | 2721 | 555 | 1.51 (1.26, 1.82) | <0.001 |
| Mouth/Tongue/Lip Cancer | 1706 | 478 | 1.24 (1.08, 1.42) | 0.002 |
| Bladder Cancer | 4704 | 1241 | 1.59 (1.43, 1.77) | <0.001 |
| Cervical Cancer | 440 | 108 | 0.83 (0.64, 1.08) | 0.167 |
| Testicular Cancer | 361 | 42 | 2.77 (1.98, 3.87) | <0.001 |
| Rectal Cancer | 4461 | 1467 | 1.08 (0.98, 1.20) | 0.127 |
| Colon Cancer | 7077 | 2362 | 1.05 (0.96, 1.16) | 0.298 |
| Lymphoma/Hodgkin's Disease | 4287 | 1390 | 1.05 (0.94, 1.16) | 0.398 |
| Soft Tissue Cancer | 667 | 238 | 0.91 (0.77, 1.07) | 0.236 |
| Kidney Cancer | 2541 | 930 | 0.93 (0.83, 1.03) | 0.172 |
| Larynx/Windpipe Cancer | 796 | 353 | 0.85 (0.74, 0.99) | 0.031 |
| Leukemia | 2648 | 1150 | 0.75 (0.67, 0.83) | <0.001 |
| Ovarian Cancer | 1976 | 982 | 0.45 (0.37, 0.54) | <0.001 |
| Bone Cancer | 273 | 116 | 0.66 (0.54, 0.82) | <0.001 |
| Other Cancer | 8669 | 4658 | 0.52 (0.48, 0.57) | <0.001 |
| Blood Cancer | 1907 | 782 | 0.80 (0.71, 0.90) | <0.001 |
| Lung Cancer | 6316 | 4362 | 0.35 (0.32, 0.39) | <0.001 |
| Stomach Cancer | 1461 | 960 | 0.38 (0.34, 0.42) | <0.001 |
| Esophageal Cancer | 1933 | 1293 | 0.37 (0.34, 0.41) | <0.001 |
| Pancreatic Cancer | 1992 | 1668 | 0.20 (0.18, 0.23) | <0.001 |
| Liver Cancer | 1151 | 899 | 0.26 (0.23, 0.29) | <0.001 |
| Brain Cancer | 1192 | 995 | 0.17 (0.15, 0.19) | <0.001 |
| Gallbladder Cancer | 183 | 160 | 0.15 (0.12, 0.18) | <0.001 |
| Melanoma | / | / | / | / |
| Nervous System Cancer | 93 | 38 | 0.67 (0.48, 0.94) | 0.021 |
| Non-Melanoma Skin Cancer | / | / | / | / |
| Unknown Skin Cancer | / | / | / | / |

Models were adjusted for age ,sex, and body mass index. Squares represent HR estimates, and horizontal lines represent 95%CIs.

**Supplementary Table 10. Primary cause of Mortality in patients with diabetic foot disease in the NHANES cohorts.**

| **Primary cause of death** | **Cancer** | **Diabetes** | **Diabetic Foot** Disease |
| --- | --- | --- | --- |
| All-cause mortality, N (%) | 744 (61.0) | 258 (41.4) | 410 (71.7) |
| Cardiovascular and cerebrovascular diseases, N (%) | 203 (16.7) | 75 (12.0) | 165 (28.8) |
| Malignant neoplasms, N (%) | 231 (18.9) | 40 (6.4) | 52 (9.1) |
| Diseases of the respiratory system, N (%) | 52 (4.3) | 13 (2.1) | 17 (3.0) |
| Diabetes mellitus, N (%) | 9 (0.7) | 28 (4.5) | 48 (8.4) |
| Influenza and pneumonia, N (%) | 18 (1.5) | 8 (1.3) | 10 (1.7) |
| Accidents (unintentional injuries), N (%) | 15 (1.2) | 5 (0.8) | 6 (1.0) |
| Diseases of the genitourinary system, N (%) | 12 (1.0) | 9 (1.4) | 23 (4.0) |
| Alzheimer’s disease, N (%) | 45 (6.0) | 10 (3.9) | 9 (2.2) |
| All other causes, N (%) | 159 (13.0) | 70 (11.2) | 80 (14.0) |

In the NHANES cohort, the primary causes of death were grouped into 10 categories.

**Supplementary Table 11. Primary cause of Mortality in patients with diabetic foot disease in the UK Biobank cohorts.**

| **Primary cause of death** | **Cancer** | **Diabetes** | **Diabetic Foot Disease** |
| --- | --- | --- | --- |
| All-cause mortality, N (%) | 25649 (25.5) | 4116 (11.4) | 687 (40.6) |
| Cardiovascular and cerebrovascular diseases, N (%) | 1828 (1.8) | 1789 (5.0) | 289 (17.1) |
| Malignant neoplasms, N (%) | 20402 (20.3) | 129 (0.4) | 5 (0.3) |
| Diseases of the respiratory system, N (%) | 802 (0.8) | 505 (1.4) | 89 (5.3) |
| Diseases of the nervous system, N (%) | 513 (0.5) | 278 (0.8) | 22 (1.3) |
| Certain infectious and parasitic diseases, N (%) | 111 (0.1) | 60 (0.2) | 9 (0.5) |
| Diseases of the digestive system, N (%) | 461 (0.5) | 325 (0.9) | 38 (2.2) |
| External causes of morbidity and mortality, N (%) | 216 (0.2) | 143 (0.4) | 13 (0.8) |
| Codes for special purposes, N (%) | 452 (0.4) | 319 (0.9) | 30 (1.8) |
| Diseases of the skin and subcutaneous tissue, N (%) | 17 (0.0) | 15 (0.0) | 8 (0.5) |
| Diseases of the genitourinary system, N (%) | 96 (0.1) | 60 (0.2) | 26 (1.5) |
| Diseases of the musculoskeletal system and connective tissue, N (%) | 64 (0.1) | 29 (0.1) | 6 (0.4) |
| Endocrine, nutritional and metabolic diseases, N (%) | 102 (0.1) | 188 (0.5) | 107 (6.3) |
| All other causes, N (%) | 2105 (2.1) | 1415 (3.9) | 282 (16.7) |
| Symptoms, signs and abnormal clinical and laboratory findings, not elsewhere classified, N (%) | 26 (0.0) | 27 (0.1) | 1 (0.1) |

In the UKB cohort, the primary causes of death were grouped into 14 categories.
